# Treatment of Trismus and Tetanus by Hydrate of Chloral

**Published:** 1873-07

**Authors:** 


Treatment of Trismus and Tetanus by Hydrate of Chloral. Dr. Th. Kœhler, of Kassel, Germany, reports a case of intense trismus, tetanus, opisthotonos, in consequence of a gunshot wound of the elbow-joint, received by a German soldier in the battle of Beaugency, on the 10th of December, 1870. On the 18th of December the first symptoms of the disease appeared, and within a few days the above named condition existed in a high degree, with severe attacks of spasms and dyspnœa. Thirty grain doses of hydrate of chloral, twice daily, were given from the 24th day of December to the 6th of January, with a perceptible amelioration of the symptoms. On the latter day, however, a very severe attack of spasms, with complete and firm closure of the jaws, and entire unconsciousness gave occasion to use thirty grains of the above named medicine, four times daily for two days; but as no decided relief of the tetanus was obtained, on the third day sixty grains of the remedy were given twice. A deep and long sleep followed, with a decided relief of all symptoms, and the patient continued to improve daily without any more medicine, and was discharged well at the end of January.—*Berliner Klinische Wochenschrift*, *No.* 5, 1873.

